# Child temperament and early childhood caries: is there a link?

**DOI:** 10.1038/s41432-024-01057-9

**Published:** 2024-09-03

**Authors:** Joshua Kennedy, Greig Taylor

**Affiliations:** 1 Academic Dental Foundation Trainee, School of Dental Sciences, Newcastle University, Faculty of Medical Science, Framlington Place, Newcastle, NE2 4BW UK; 2 Clinical Lecturer in Paediatric Dentistry, School of Dental Sciences, Newcastle University, Faculty of Medical Science, Framlington Place, Newcastle, NE2 4BW UK

**Keywords:** Dental caries, Paediatric dentistry

## Abstract

**Data sources:**

A systematic search was conducted across multiple databases (MEDLINE via PubMed, EMBASE, Scopus, LILACS, Web of Science, and EBSCO) up to January 2023.

**Study selection:**

Any case-control, cohort, or cross-sectional study which assessed child temperament and early childhood caries (ECC) in children aged six years or younger were included. Literature reviews, studies with insufficient data, non-English publications, and those focusing on older children or adults were excluded.

**Data extraction and synthesis:**

Data extraction was conducted independently by two authors, with a third author resolving any disagreements. Risk of bias was assessed using the Newcastle-Ottawa assessment scale (case-control and cohort studies) and the Appraisal tool for Cross-Sectional Studies (cross-sectional studies). The quality of evidence was evaluated using the Grading of Recommendations, Assessment, Development, and Evaluations (GRADE) approach. Statistical analysis to evaluate heterogeneity included the chi-square test and the I-square index.

**Results:**

A total 5072 studies resulted in the inclusion of 15 studies, encompassing data from 6,667 participants. Seven studies were of high quality and eight, moderate. Meta-analyses of seven studies revealed a significant association between certain temperament traits (e.g., higher levels of emotionality and lower levels of sociability) and ECC. In particular, difficult temperament was associated with ECC (OR 2.63 95%CI: 1.37–5.04)

**Conclusions:**

The study concluded that child temperament is a significant factor in the risk of developing ECC. Specifically, children with higher emotionality and lower sociability are at greater risk. Interventions targeting child temperament through child behaviour and parental management strategies may be effective in reducing ECC.

## A Commentary on

**Sriram S, Sahoo S, Muthu M S** **et al**.

Child Temperament and Early Childhood Caries: A Systematic Review and Meta-Analysis. *Pediatr Dent* 2024; **46:** 169–178.

**GRADE Rating:** 

## Commentary

Early childhood caries (ECC) is highly prevalent and remains a significant public health issue for many young children worldwide^1,2^. ECC is a destructive form of dental caries that manifests early in life^3^. It is multifactorial, arising from the interaction of cariogenic microorganisms, exposure to fermentable carbohydrates, and various social determinants, including inappropriate feeding practices and socioeconomic conditions^3–5^. The consequences of ECC are far-reaching, affecting the oral health, overall well-being and development of children. It is preventable, or arrestable, if conventional oral health strategies are adopted. However, children who have behavioural or temperament issues might struggle to adhere to such strategies, increasing their risk of ECC development^2^.

Child temperament refers to the constitutionally based differences in behavioural style and predisposition that are observable from early infancy. Temperament encompasses individual differences in both reactivity and self-regulation. Reactivity is the ease with which a child experiences motor and affective arousal, such as fear, anger, and sadness. Regulation, or effortful control, is the ability to manage and modulate this reactivity. Effortful control involves the capacity to inhibit impulses, maintain attention on tasks, and resist distractions^6^.

Common scales for assessing temperament include the Emotionality, Activity, and Sociability (EAS) Temperament Survey for Children, Thomas and Chess’s Parent Temperament Questionnaire, the Malhotra Temperament Scale (MTS), the Early Childhood Behaviour Questionnaire (ECBQ), its Short Form, and Very Short Form (ECBQ-VSF), and Cloninger’s Preschool Temperament and Character Inventory (74-item questionnaire)^7^. Some of these questionnaires, particularly the ECBQ, have faced criticism. The ECBQ relies on parental reports, which may be influenced by social desirability bias, leading to overly positive portrayals of children. Koning et al. highlight significant discrepancies between parent and child reports of health-related behaviours, suggesting that parents may not fully understand their child’s behaviour in non-home settings^8^. To improve accuracy, Koning et al. recommend including observations from both parents and childcare providers to capture a more complete picture of a child’s temperament across different environments^8^.

The authors of this study suggest a possible link between ECC and child temperament. Temperament is a complex factor influenced by genetic, environmental, socio-economic as well as cultural elements, making it challenging to isolate its effects. While generally stable and present from birth, temperament varies among individuals and can be shaped by environmental influences throughout life^6^.

This review explores if there is a link; however, it leaves a lot of unanswered questions due to the paucity of high-quality studies. Establishing a definitive link may necessitate longitudinal studies to observe long-term effects. However, these studies are resource-intensive and time-consuming. Addressing the main concern in any future study would be to eliminate confounding factors or acknowledging them and analysing the data accordingly to account for them e.g., regression analyses. Despite these perceived future methodological challenges, exploring this connection remains important for understanding and managing ECC in young children.

Despite growing concerns about systematic reviews which conclude that further studies are needed, it did seem relevant to conduct this review  as the link between child temperament and ECC makes sense and has not been formally assessed. Given the strict inclusion/exclusion criteria, and limited studies available for meta-analysis, a scoping- or umbrella review might have been more pertinent. However, this systematic review was conducted in a robust manner, adhering strictly to the PRISMA guidelines^9^, which allows the reader to interpret the results with a high degree of confidence. A comprehensive search was undertaken and included a wide range of studies from multiple database searches. This was a particular strength of the study as there is risk in systematic reviews that only studies from the main databases e.g., PubMed, are included. Additional searching of grey literature would have been favourable. Independent data extraction by two researchers, with disagreements resolved by a third, reduced subjective bias. Assessment of the overall quality and risk of bias was undertaken using appropriate tools. Appropriate statistical methods, such as the chi-square test and I-square index, were used to evaluate heterogeneity among the studies, ensuring that variability did not skew results. Although these should be expected in a review, it is not always the case, so the concerted efforts by the authors really do highlight to the reader that a detailed and thorough approach has been undertaken.

There were some limitations, however, the authors did transparently report them. They key issues being heterogeneity amongst most of the included studies. Although standardised measures to assess temperament and ECC in the individual studies were used, these were not consistent. Adopting a core-outcome set could support reduced heterogeneity in future reviews. Similarly, excluding non-English publications might have omitted relevant studies.

It is accepted that oral health education and interventions can significantly reduce ECC^10^. Spitz et al. found that “difficult” children may resist toothbrushing, increasing the risk of dental decay^11^.

Early dental intervention is vital for children under six, as early caries in primary teeth often predict future dental issues. While the World Health Organization focuses on school-aged children, this research advocates for earlier preventive measures^2^. Clinicians should provide anticipatory guidance tailored to a child’s temperament, offering specific advice for those with negative or shy temperaments. Interventions may include improving oral hygiene practices and reducing sugary food and drink intake. The approach may vary depending on a child’s temperament and dental anxiety, as well as cultural differences and parenting practices. However, highlighting the need to account for temperament early will benefit both the child and their family.

It does appear that child temperament and ECC are intimately linked as children with negative temperaments do appear to be at higher risk for ECC. Awareness of this relationship is important for clinicians to support children and their families given the long-term impacts ECC can have.

Practice points

- **Holistic Assessment:** Incorporate behavioural assessments into routine check-ups to identify children at higher risk for ECC.
- **Parental Guidance:** Educate parents on the impact of child temperament on oral health and provide strategies for managing challenging behaviours.
- **Targeted Interventions:** Develop tailored preventive measures for children with high emotionality and low sociability.
- **Interdisciplinary Approach:** Collaborate with other healthcare professionals to address both dental and behavioural health.
- **Ongoing Research:** Encourage further research to explore the relationship between temperament and ECC and identify effective intervention strategies.
